# Supplementary material for: Point-of-care HIV viral load testing in a community antiretroviral therapy delivery programme: A randomised controlled trial (PHILA)
Source: PLOS Glob Public Health. 2026 Mar 2;6(3):e0005890. doi: 10.1371/journal.pgph.0005890 (PMC12952611; doi:10.1371/journal.pgph.0005890)
Supplement: S1 Text — (DOCX) [file pgph.0005890.s001.docx]

**S1 Text: Figures and tables for Point-of-care HIV viral load testing in a community antiretroviral therapy delivery programme: a randomised controlled trial (PHILA)**

**Contents**

[Fig A: Process maps of viral load testing in the PHILA trial 2](#_Toc219724163)

[Fig B: Time to receipt of viral load results in the PHILA trial 5](#_Toc219724164)

[Fig C: Numbers of CCMDD prescriptions, and their cycle lengths, by month in the PHILA trial 6](#_Toc219724165)

[Fig D: Time to ART collection in CCMDD in the PHILA trial 7](#_Toc219724166)

[Table A: Secondary outcomes before and after the guideline change to allow immediate renewal of CCMDD prescriptions without review of viral load results 9](#_Toc219724167)

[Table B: Reasons for not collecting antiretroviral therapy in the PHILA trial, n = 24 9](#_Toc219724168)

[Table C: Characteristics of people receiving a renewed referral to CCMDD at the time of renewed referral in 108 facilities between 24^th^ May and 10^th^ September, n = 16,568 9](#_Toc219724169)

## Fig A: Process maps of viral load testing in the PHILA trial


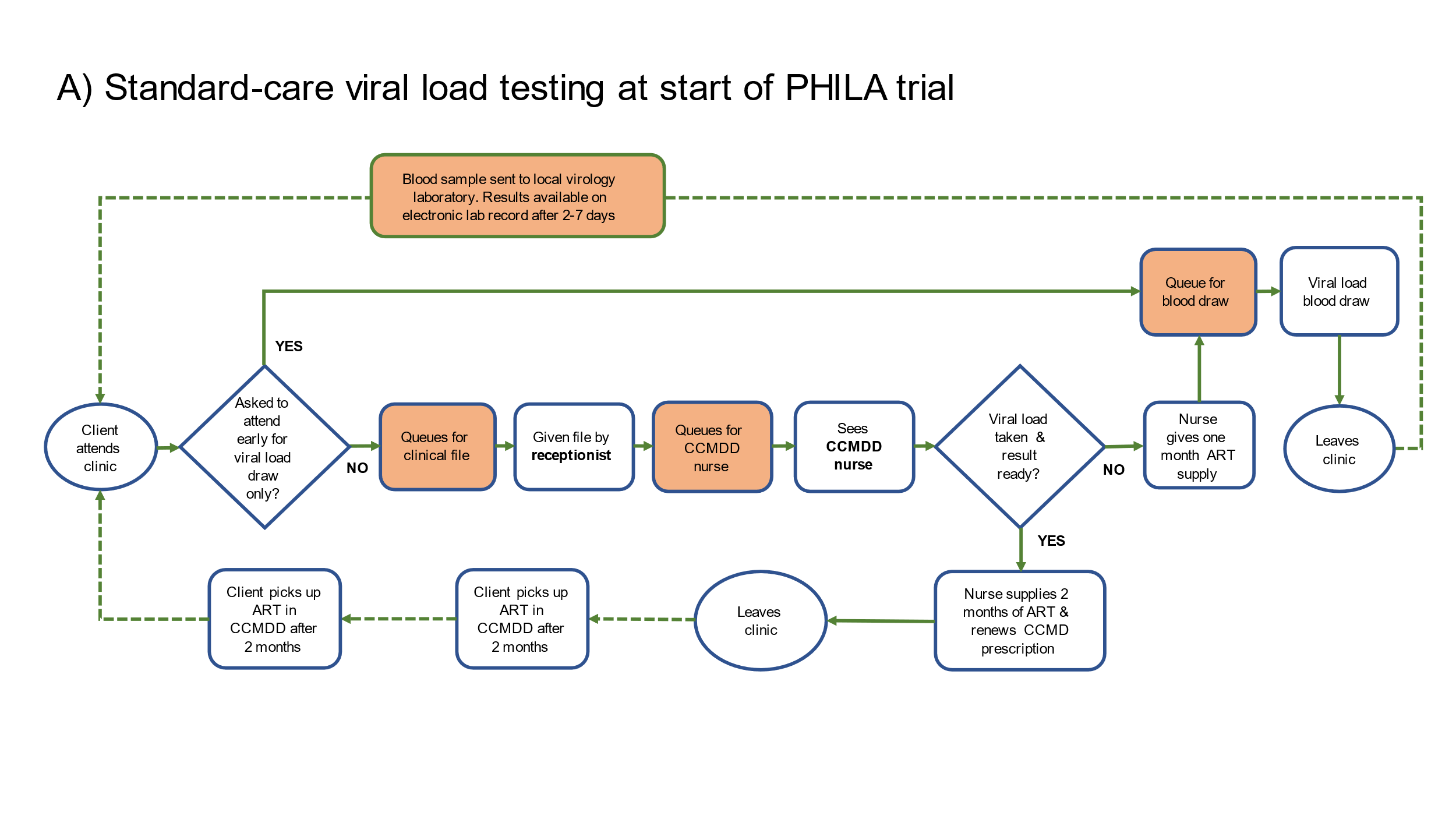


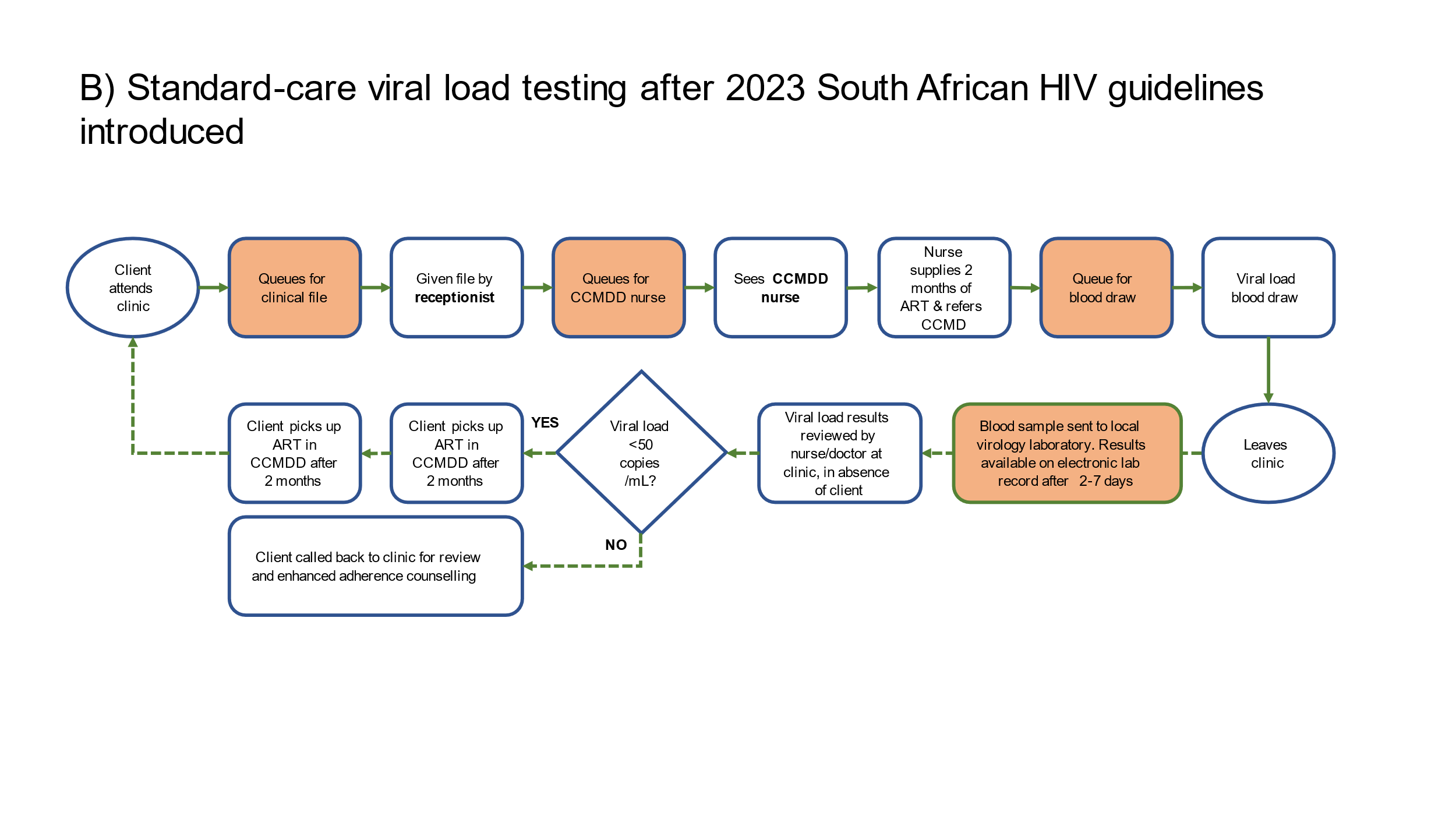


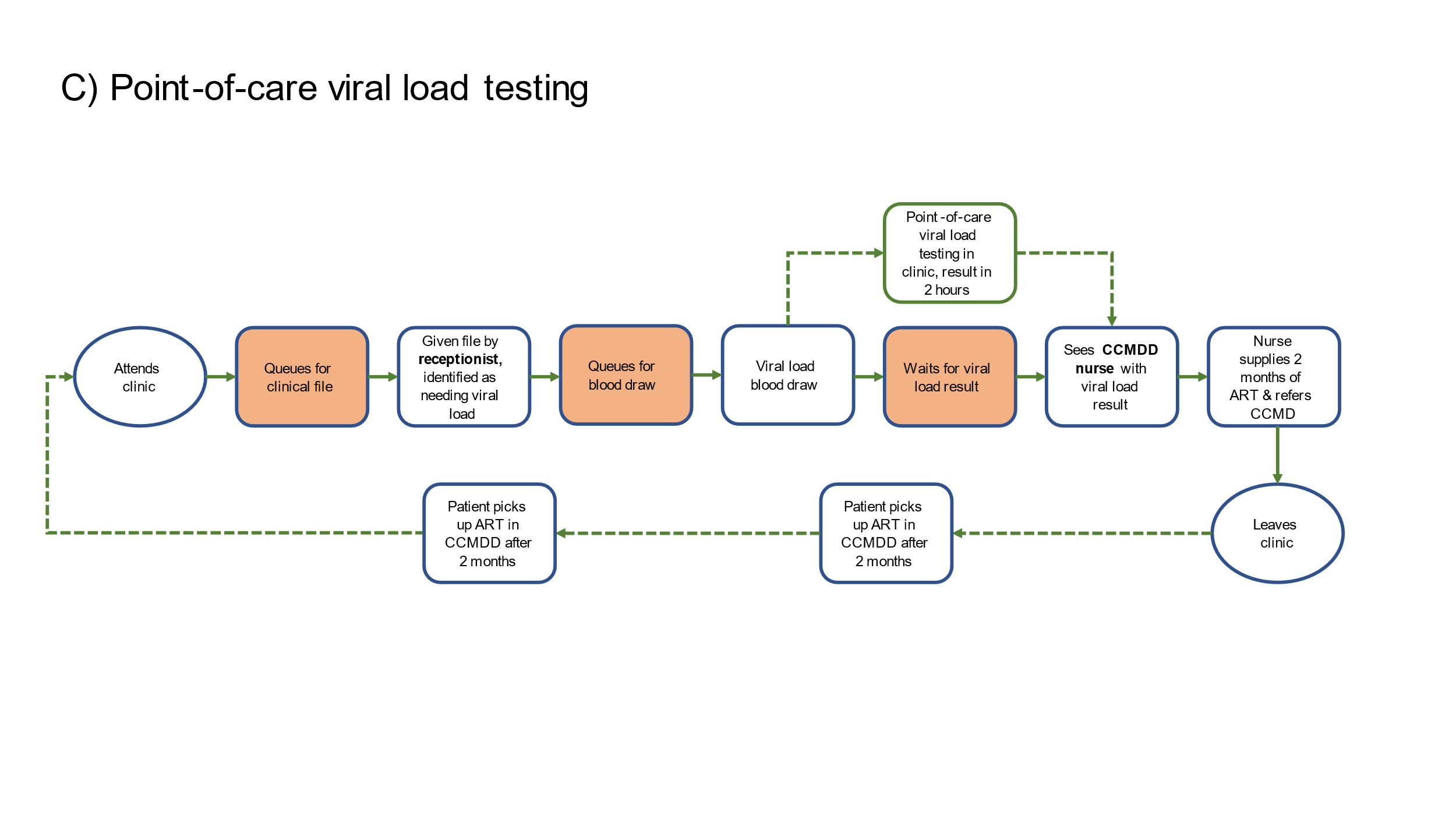


## Fig B: Time to receipt of viral load results in the PHILA trial


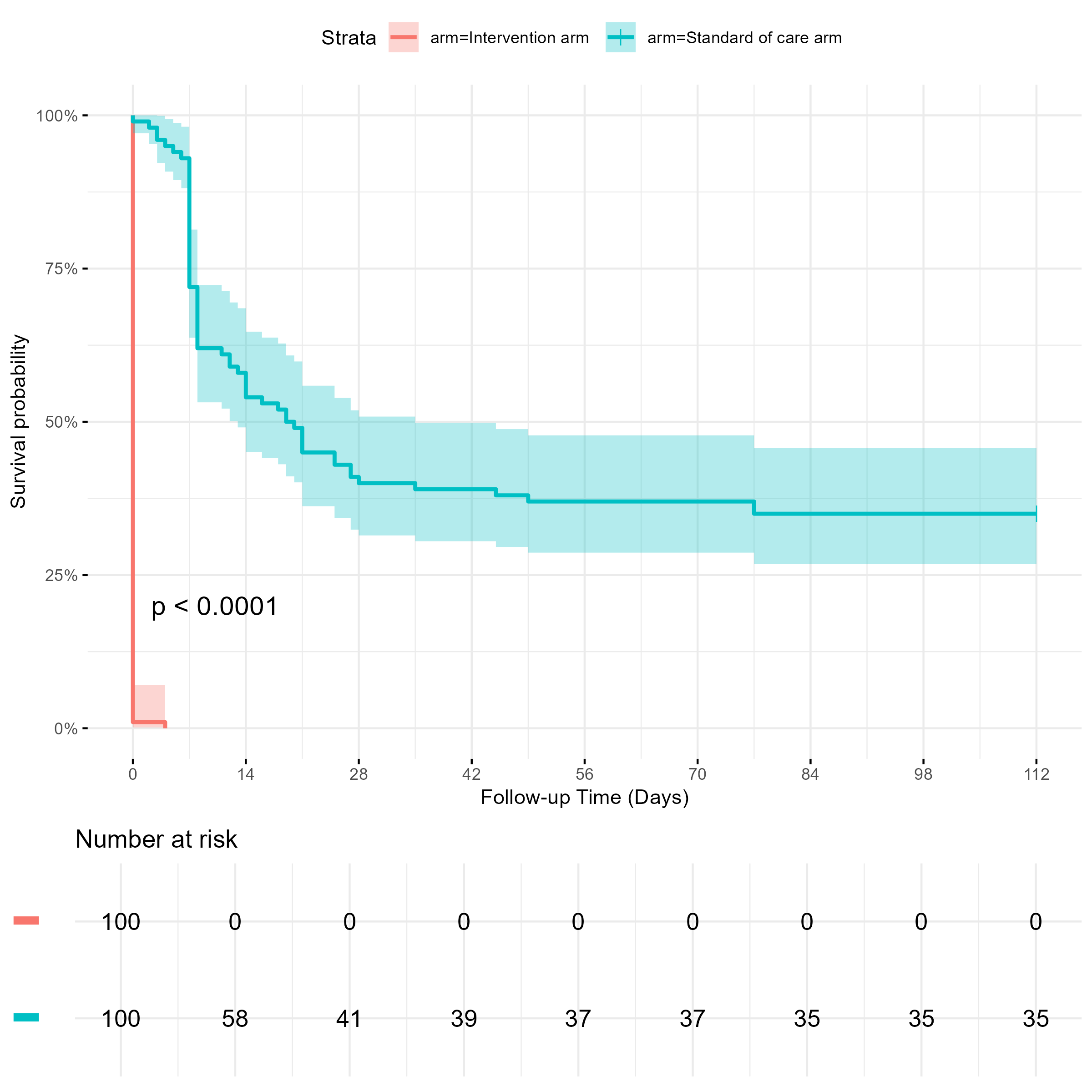


## Fig C: Numbers of CCMDD prescriptions, and their cycle lengths, by month in the PHILA trial


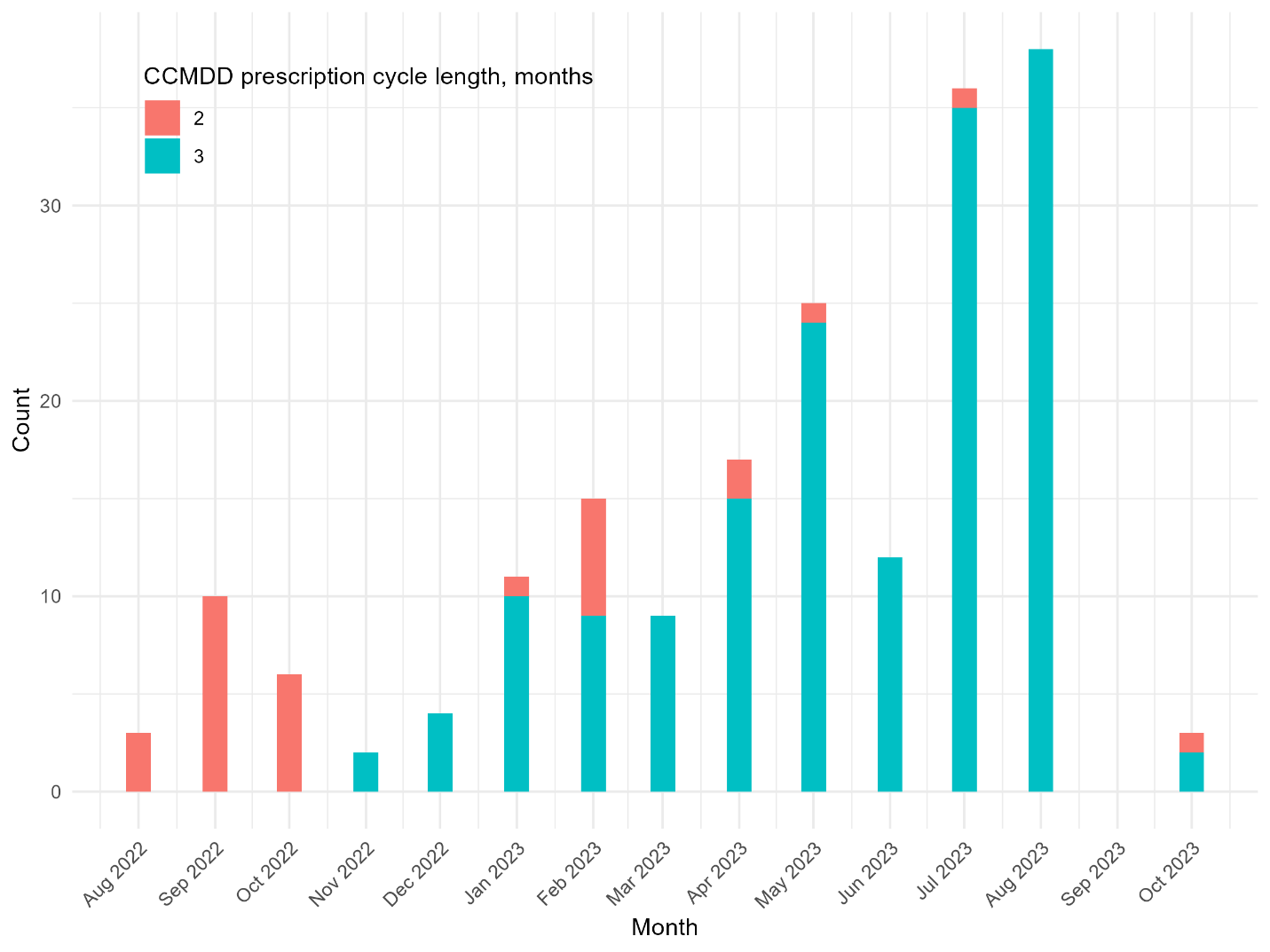


## Fig D: Time to ART collection in CCMDD in the PHILA trial


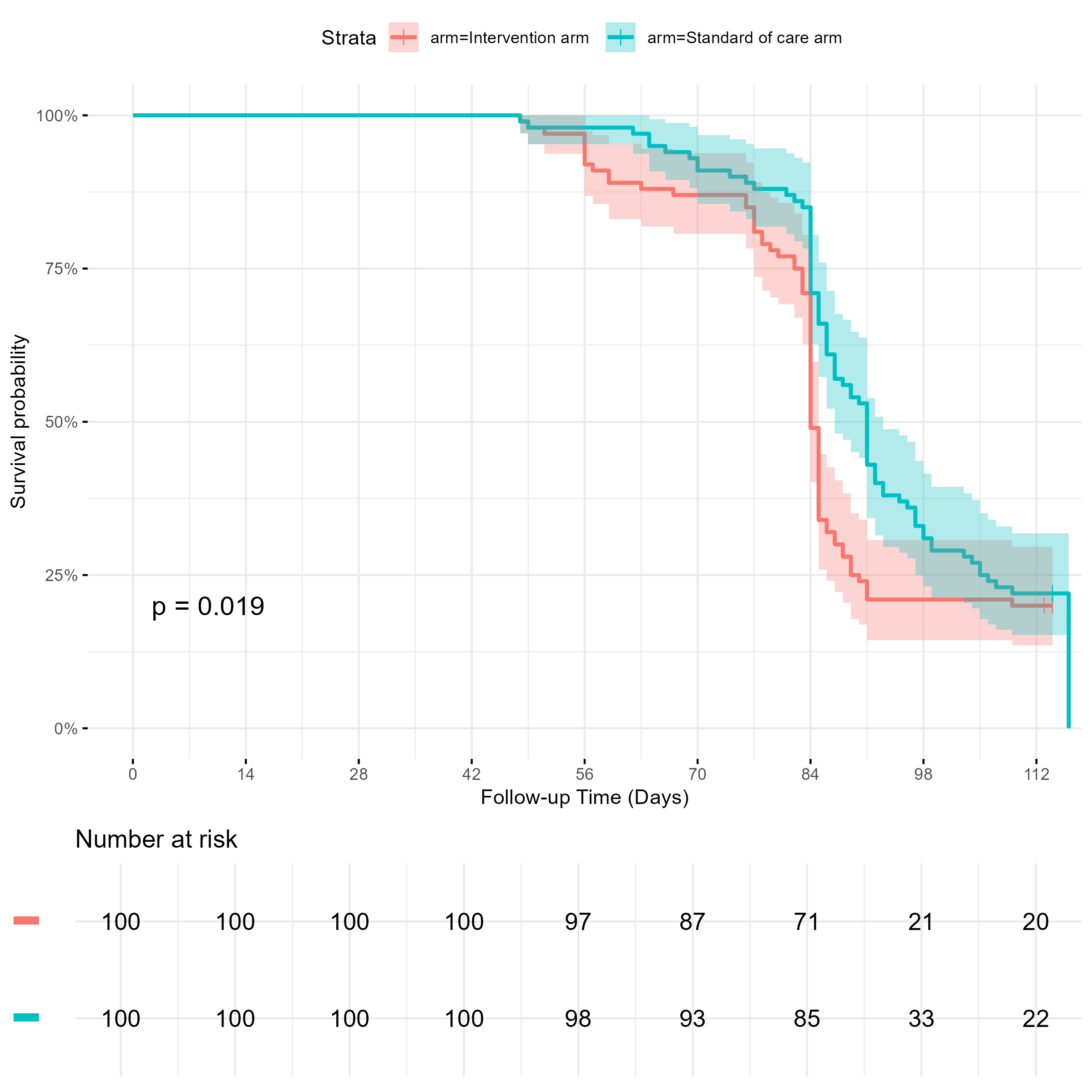


## Table A: Secondary outcomes before and after the guideline change to allow immediate renewal of CCMDD prescriptions without review of viral load results

|  | **Intervention**  **n/N (%)** | **Standard care**  **n/N (%)** | **Risk difference**  **(95% CI)** | **p value** |
| --- | --- | --- | --- | --- |
| **Secondary outcomes** |  |  |  |  |
| Time to participant receiving enrolment VL result, days (median, IQR) | 0 (0, 0) | 20 (7, Not received) | - | - |
| Before guideline change | 0 (0, 0) | 8 (7, 21) | - | - |
| After guideline change | 0 (0, 0) | Not received (19, Not received) | - | - |
| Time to CCMDD prescription renewal, days (median, IQR) | 0 (0, 0) | 7 (0, 12) | - | - |
| Before guideline change | 0 (0, 0) | 7 (8, 21) | - | - |
| After guideline change | 0 (0, 0) | 0 (0, 6) | - | - |
| Time to first CCMDD ART collection, days (median, IQR) | 84 (83 to 89) | 91 (84 to 105) | - | - |
| Before guideline change | 84 (74 to 86) | 95 (88 to 112) | - | - |
| After guideline change | 85 (84 to 91) | 86 (84 to 91) | - | - |
| Proportion of participants retained-in-care | 89/100 (89.0%) | 87/100 (87.0%) | 2.0% (-8.0 to 12.0) | 0.828 |
| Before guideline change | 47/52 (90.4%) | 44/51 (86.3%) | 4.1% (-10.2 to 18.4) | 0.732 |
| After guideline change | 42/48 (87.5%) | 43/49 (87.8%) | -0.3% (-13.6 to 13.1) | 1.00 |
|  | **Mean (95% CI)** | **Mean (95% CI)** | **Mean difference (95% CI)** |  |
| Number of clinic visits required for CCMDD renewal | 1.06 (1.00 - 1.12) | 1.60 (1.47 -1.73) | -0.54 (-0.40 to -0.68) | <0.001 |
| Before guideline change | 1.00 (1.00 to 1.00) | 1.88 (1.74 to 2.03) | -0.88 (-0.74 to -1.03) | <0.001 |
| After guideline change | 1.12 (1.00 to 1.25) | 1.31 (1.13 to 1.48) | -0.19 (-0.40 to 0.04) | 0.041 |
| Travel costs from the patient perspective to have CCMDD prescription renewed (ZAR) | 47.7 (39.6 – 55.8) | 72.8 (59.0 – 86.7) | -25.1 (-9.2 to -41.1) | <0.001 |
| Before guideline change | 38.2 (33.4 to 43.1) | 78.1 (63.7 to 92.5) | -39.9 (-24.7 to -55.0) | <0.001 |
| After guideline change | 58.0 (42.1 to 73.8) | 67.3 (42.9 to 91.8) | -9.3 (-38.2 to 19.4) | 0.184 |

## Table B: Reasons for not collecting antiretroviral therapy in the PHILA trial, n = 24

| **Description** | **N** |
| --- | --- |
| Participant not contacted | 13 |
| Participant travelling so couldn’t collect | 4 |
| Participant reported they had collected package, although no record of collection in SYNCH | 3 |
| Participant did not find antiretroviral therapy package at community pickup point | 1 |
| Participant died | 1 |
| Participant stated had enough ART so did not need to collect | 1 |
| Participant did not receive SMS reminder and so forgot to collect ART | 1 |

## Table C: Characteristics of people receiving a renewed referral to CCMDD at the time of renewed referral in 108 facilities between 24^th^ May and 10^th^ September, n = 16,568

| **Variable** | **Levels** | **Overall**  **(n=16,568; column %)** | **Viral load at referral ≤50 copies/mL**  **(n=13,636; row %)** | **Viral load at referral >50 copies/mL**  **(n=2,932; row %)** |
| --- | --- | --- | --- | --- |
| **Demographics** | | | | |
| Age, years | Median (IQR) | 41 (35 to 48) | 41 (35 to 48) | 42 (37 to 49) |
| Gender | Female | 11,477 (69.3%) | 9,558 (83.3%) | 1,919 (16.7%) |
|  | Male | 5,091 (30.7%) | 4,078 (80.1%) | 1,013 (19.9%) |
| Region | eThekwini Metropolitan Municipality | 12,734 (76.9%) | 10,012 (78.6%) | 2,722 (21.4%) |
|  | uMgungundlovu District Municipality | 3,834 (23.1%) | 3,624 (94.5%) | 210 (5.5%) |
| **Clinical information** | | | | |
| Time since ART initiation, years | Median (IQR) | 7.9 (5.1 to 10.5) | 7.9 (5.1 to 10.3) | 8.0 (5.5 to 11.0) |
| Initiation CD4 count, cells/µL^1^ | Median (IQR) | 280 (160 to 440) | 290 (170 to 450) | 250 (140 to 370) |
| Initiation CD4 count category, cells/µL^1^ | <200 | 4,059 (32.0%) | 3,190 (78.6%) | 869 (21.4%) |
|  | 200-349 | 3,899 (30.7%) | 3,128 (80.2%) | 771 (19.8%) |
|  | 350-499 | 2,373 (18.7%) | 1,984 (83.6%) | 389 (16.4%) |
|  | >=500 | 2,360 (18.6%) | 2,067 (87.6%) | 293 (12.4%) |
|  | (Missing) | 3,877 | 3,267 | 610 |
| Current ART regimen at enrolment | TDF / XTC / DTG | 15,640 (94.4%) | 12,915 (82.6%) | 2,725 (17.4%) |
|  | TDF / XTC / EFV | 303 (1.8%) | 255 (84.2%) | 48 (15.8%) |
|  | Other | 625 (3.8%) | 466 (74.6%) | 159 (25.4%) |
| Time on current regimen, years | Median (IQR) | 2.5 (1.8 to 3.0) | 2.5 (1.7 to 3.0) | 2.5 (1.8 to 3.0) |
| Time since first CCMDD referral, years | Median (IQR) | 3.8 (1.8 to 5.6) | 3.8 (1.8 to 5.7) | 3.6 (1.8 to 5.5) |
| Time since latest CCMDD referral, days | Median (IQR) | 168 (168 to 172) | 168 (168 to 172) | 168 (168 to 172) |
| Time since previous viral load, days | Median (IQR) | 358 (335 to 378) | 360 (336 to 378) | 350 (286 to 379) |
| Previous viral load result, copies/mL | ≤50 | 14,985 (90.8%) | 12,618 (84.2%) | 2,367 (15.8%) |
|  | 51 - 999 | 1,443 (8.7%) | 918 (63.6%) | 525 (36.4%) |
|  | ≥1000 | 80 (0.5%) | 45 (56.3%) | 35 (48.3%) |
|  | (Missing) | 60 | 55 | 5 |

^1^ Closest to ART initiation, up to 180 days before and up to 30 days after ART initiation.
