# Supplementary material for: Point-of-care HIV viral load testing in a community antiretroviral therapy delivery programme: A randomised controlled trial (PHILA)
Source: PLOS Glob Public Health. 2026 Mar 2;6(3):e0005890. doi: 10.1371/journal.pgph.0005890 (PMC12952611; doi:10.1371/journal.pgph.0005890)
Supplement: Attachment — Submitted filename: Response to Reviewers-R1_PGPH_FINAL.docx [file pgph.0005890.s004.docx]

25^th^ November 2025

Dear Dr Megan Coffee and colleagues,

We are grateful to you and the reviewers for the opportunity to submit a revised version of our manuscript, “**Point-of-care HIV viral load testing in a community antiretroviral therapy delivery programme: a randomised controlled trial (PHILA)**”, to *PLOS Global Public Health.* We attach a point-by-point response to the reviewers’ comments. Line numbers are for the marked-up copy of the revised manuscript:

**Journal Requirements:**

**Comment 1**. Please provide a detailed online Financial Disclosure statement. This is published with the article. It must therefore be completed in full sentences and contain the exact wording you wish to be published.

a) Please clarify all sources of financial support for your study. List the grants, grant numbers, and organizations that funded your study, including funding received from your institution. Please note that suppliers of material support, including research materials, should be recognized in the Acknowledgements section rather than in the Financial Disclosure.

b) State the initials, alongside each funding source, of each author to receive each grant. For example: “This work was supported by the National Institutes of Health (####### to AM; ###### to CJ) and the National Science Foundation (###### to AM).”

c) State what role the funders took in the study. If the funders had no role in your study, please state: “The funders had no role in study design, data collection and analysis, decision to publish, or preparation of the manuscript.”

**Response:** Done

**Comment** 2. Please ensure that the funders and grant numbers match between the Financial Disclosure field and the Funding Information tab in your submission form. Note that the funders must be provided in the same order in both places as well.

**Response:** Done

**Comment** 3. In the online submission form, you indicated that “Bona fide researchers will be able to request access to anonymised trial data by contacting the corresponding author.”.

All PLOS journals now require all data underlying the findings described in their manuscript to be freely available to other researchers, either

a) In a public repository,

b) Within the manuscript itself, or

c) Uploaded as supplementary information.

This policy applies to all data except where public deposition would breach compliance with the protocol approved by your research ethics board. If your data cannot be made publicly available for ethical or legal reasons (e.g., public availability would compromise patient privacy), please explain your reasons by return email and your exemption request will be escalated to the editor for approval. Your exemption request will be handled independently and will not hold up the peer review process, but will need to be resolved should your manuscript be accepted for publication. One of the Editorial team will then be in touch if there are any issues.

**Response:** The approved protocol does not provide for making the underlying data freely available to other researchers, and neither do we have patient consent for this. However, we do have approval for “de-identified data to be shared with other researchers in South Africa, the UK or the United States of America for research purposes”. Therefore, we will need to determine where the researchers are, and for what purpose they are requesting the data, before sharing it. We have communicated this by email with the editorial team.

**Comment** 4. Please provide separate main figure files in .tif or .eps format only and ensure that all files are under our size limit of 10MB.

For more information about how to convert your figure files please see our guidelines: https://journals.plos.org/globalpublichealth/s/figures

**Response:** Done

**Comment** 5. Please ensure that you refer to Figure 1 in your text as, if accepted, production will need this reference to link the reader to the figure.

**Response:** Figure 1 is mentioned on line 251

**Comment 6**. Please include a separate legend or caption for Figure 1 in your manuscript.

**Response:** Done

**Comment 7**. We notice that your supplementary figures and tables are included in the manuscript file. Please remove them and upload them with the file type 'Supporting Information'. Please ensure that each Supporting Information file has a legend listed in the manuscript before or after the references list.

**Response:** Done

**Comment 8**. We have noticed that you have uploaded Supporting Information files, but you have not included a list of legends. Please add a full list of legends for your Supporting Information files before or after the references list.

**Response:** Done

**Comment 9**. Some material included in your submission may be copyrighted. According to PLOS’s copyright policy, authors who use figures or other material (e.g., graphics, clipart, maps) from another author or copyright holder must demonstrate or obtain permission to publish this material under the Creative Commons Attribution 4.0 International (CC BY 4.0) License used by PLOS journals. Please closely review the details of PLOS’s copyright requirements here: PLOS Licenses and Copyright. If you need to request permissions from a copyright holder, you may use PLOS's Copyright Content Permission form.

Please respond directly to this email or email the journal office and provide any known details concerning your material's license terms and permissions required for reuse, even if you have not yet obtained copyright permissions or are unsure of your material's copyright compatibility.

Potential Copyright Issues:

‘Figure 1’ and ‘Figure 2’ in “PHILA_POC_VL_CCMDD_Protocol_Version_2.0_10Nov21_clean.pdf”: Please confirm whether you drew the images / clip-art within the figure panels by hand. If you did not draw the images, please provide (a) a link to the source of the images or icons and their license / terms of use; or (b) written permission from the copyright holder to publish the images or icons under our CC-BY 4.0 license. Alternatively, you may replace the images with open source alternatives. See these open source resources you may use to replace images / clip-art:

- https://commons.wikimedia.org

- https://openclipart.org/

**Response:** We have removed these images

**Comment 10**. If the reviewer comments include a recommendation to cite specific previously published works, please review and evaluate these publications to determine whether they are relevant and should be cited. There is no requirement to cite these works unless the editor has indicated otherwise.

**Response**: Done

**Reviewers' comments:**

Reviewer's Responses to Questions

Comments to the Author

**Reviewer #1:**

General comments:

This manuscript reports the results of an open-label, randomized controlled trial of point-of-care HIV viral load (VL) testing versus laboratory-based VL testing on antiretroviral therapy (ART) prescription renewal proportions among people living with HIV in Durban, South Africa.

**Comment 1**: I have a couple of general comments about the analytic approach for this trial. In expectation, randomization will "balance" the treatment groups of a randomized trial. As is often the case with a randomized study, many variables have similar distributions in the treatment groups, but there are a few that differ. Given your sample size, I believe you could also control for some of these covariates that differ between groups. I would encourage moving to a multivariable model to achieve this. The choice of method will depend on how you want to report your estimate.

**Response:** Thank you for your review and comments. The FDA recommends the covariate adjustment for covariates likely to be strongly correlated with the outcome:

<https://www.fda.gov/regulatory-information/search-fda-guidance-documents/adjusting-covariates-randomized-clinical-trials-drugs-and-biological-products>

We hypothesised that the main barrier to achieving the outcome of CCMDD renewal within 3 weeks was a health system one due to unavailability of viral load results, rather than an individual client characteristic. Other healthcare system barriers to CCMDD renewal may also play a role, such as lack of healthcare worker time/training, problems with the electronic CCMDD renewal system. We did not expect any patient level characteristics to be strongly correlated with the outcome, and so did not prespecify controlling for imbalanced baseline variables in the study protocol. Therefore, we have not adjusted the primary analysis, but for the reviewer’s interest we here present a sensitivity analysis using a Poisson regression model with robust standard errors and imbalanced variables which could (although we feel are unlikely to) be correlated with the outcome (sex, income, hazardous alcohol use, and CD4 count at ART initiation) included as co-variates. The results are not meaningfully affected.

Unadjusted absolute risk difference +12.0% (95% CI 2.8 to 21.2). Adjusted absolute difference: +16.1% (95%CI 5.7 to 26.5)

**Comment 2:** That leads to my second general comment: I believe the risk difference quantity is in percentage points and not percentage. For example, in the abstract (line 69) a percentage difference, should be ~15% since 93/81=1.148... and not 12%. Thus, you'll either need to change to percentage differences or change the reporting and language to indicate that this is a percentage point difference.

**Response:** To ensure clarity we now report absolute risk differences throughout the manuscript. We now say:

Line 216: *“In pre-specified analyses, we calculated the proportions of participants achieving study outcomes and compared proportions in each arm using the chi-squared test, and presented absolute risk differences in percentage points.”*

Line 283: *“In the intervention arm, 93/100 (93.0%) of participants had a CCMDD prescription renewal within three weeks, compared to 81/100 (81.0%) in the standard-of-care arm (absolute risk difference [RD] 12.0%, 95% confidence interval [CI] 2.9 to 21.2%, p=0.021, Table 3*”

Specific comments:

**Comment** 1. (line 99) Typo ("0")

**Response:** Corrected

**Comment** 2. (lines 147-149) What algorithm or software was used to random participants? How was the randomization implemented? What block sizes were used?

**Response:** We now clarify that:

Line 152: “*After providing written informed consent and screening, eligible participants were enrolled and randomised by a study nurse, using a pre-programmed* ***REDCap (Research Electronic Data Capture),^14^****electronic case report form, in a 1:1 ratio to the point-of-care arm or the standard-of-care arm. A statistician generated the allocation sequence using computer generated random numbers with variable block sizes. All other study staff were blinded to the block sizes and allocation sequence. Clinic staff and participants were told the participant’s allocation at enrolment.*”

**Comment** 3. (lines 204-207) What methods were used to determine power?

**Response:** We used the SampSize web app for power calculations, which we now cite here:

**Line 104: “***Assuming that 75% of participants in the standard-of-care arm would achieve the primary outcome of CCMDD renewal by 3 weeks, and a 15% improvement in the point-of-care arm, we calculated that a sample size of 100 participants per arm would give us 80% power to demonstrate superiority with a two-sided alpha of 0.05%.^15,16^”*

15. Julious SA, Campbell MJ. Tutorial in biostatistics: sample sizes for parallel group clinical trials with binary data. *Stat Med* 2012;31(24):2904-36. doi: 10.1002/sim.5381 [published Online First: 20120619]

16. Julious SA, Campbell MJ. SAMPSIZE 2012 [Available from: <https://app.sampsize.org.uk/> accessed 17 November 2025

**Comment** 4. (line 215) Please provide a methodological citation for the Newcombe-Wilson confidence interval method.

**Response:** We now cite Newcombe RG. Interval estimation for the difference between independent proportions: comparison of eleven methods. Stat Med 1998;17(8):873-90. doi: 10.1002/(sici)1097-0258(19980430)17:8<873::aid-sim779>3.0.co;2-i

**Comment** 5. (line 219) Please provide a methodological citation for your regression model.

**Response:** We now cite Zou G. A modified poisson regression approach to prospective studies with binary data. *Am J Epidemiol* 2004;159(7):702-6. doi: 10.1093/aje/kwh090

**Comment** 6. (Figure 2) I suggest changing the y-axis label to something more appropriate. You may also wish to invert the y-axis since the outcome is a positive, i.e., a higher probability is a good thing. (The same applies to Figures S3 and S4.)

**Response:** Done

**Comment** 7: (Table 1) I'd suggest including standardized mean differences in this table to allow for easier comparison of differences between the intervention and SOC arms.

**Response:** As we do not think it likely that baseline participant characteristics are associated with the outcome of CCMDD script renewal (see response to general comment 1), we have not presented the SMDs, but we do highlight large differences between the two arms.

Line 272: “*There were slightly higher proportions of women (70% versus 61%) and participants reporting no monthly income (28% versus 19%) in the intervention arm. There were more participants reporting hazardous alcohol use (76% versus 66%) in the standard-of-care arm, and the median CD4 count at initiation (308 versus 226 cells/µL) was higher in the standard-of-care arm. Other demographics and clinic variables were well balanced between the two groups.”*

**Reviewer #2:**

General comments:

This paper seeks to evaluate the impact of POC VL testing in the CCMDD renewal process in South Africa. This appears to be a well-done trial with some interesting findings regarding where POC VL did and did not have impacts. Most of the comments are just focused on getting some additional clarity on expected workflows/processes to help contextualize and interpret the varied results (and why some changed while others didn’t).

**Response:** Many thanks for your review and comments

**Comment 1:** Intro: May be helpful to contextualize VLs as a health system barrier that leads to unnecessary “dormancy”. Might also be worth briefly identifying the other reasons (e.g., client-related, treatment interruptions) or any other health system provider level.

**Response:** We now say:

Line 96: “*VLs are a potential health systems barrier to enrolment and continuation in CCMDD, as testing is conducted at centralized laboratories, meaning clients generally have to attend a clinic twice for a blood draw and then review of results, with results sometimes being delayed or lost (Figure S1A).7 This can lead to delays in renewal of CCMDD prescriptions, and increased numbers of dormant resulting in clients continuing to attend clinics rather than collecting ART in the community.*”

In line 90 we also cite papers that describe other barriers to CCMDD (refs 3-5).

**Comment 2:** Intro: I think could be worth being clear that this is also a self-inflicted problem, specifically with CCMDD continuation. It is not obvious or necessary that the health system would decide to sort of pause CCMDD until VL can be confirmed. But it is also a fact that the SA guidelines decided to do this (at least for the first part of the study). But just clarifying that specific of the SA guideline that people are expected to confirm the VL results before allowing clients to continue provides some straightforward context because it it not intuitive that this challenge would be addressed this way (I would have imagined the problem would more so be people receive CCMDD, and then poor follow up for those who end up having elevated VLs).

**Response:** We now say:

Line 93: “*Reducing the number of dormant clients has been a SANDoH priority, but has proved difficult. One potential cause is the blood tests required to determine CCMDD eligibility, as only people with a suppressed HIV viral load (VL) <50 copies/mL are eligible for enrolment into CCMDD, and prior to 2023, South African guidelines required a confirmed annual VL <1000 copies/mL for clients to have their CCMDD prescription renewed*.^7^”

**Comment 3:** Methods: Under Trial (or Control description), would state what happens regarding ART/CCMDD once VL drawn. Are clients given a one month ART supply from clinic, told to come back to clinic where VL is reviewed, and then referred back to CCMDD? Also, how does that relate to CCMDD dormany if the VL takes 3 weeks to come back? Are they then classified as dormant automatically?

**Response:** We now clarify that:

Line 32: “*Every 12 months, an annual viral load would be taken, either a few days earlier than the CCMDD prescription renewal was due (so that the result was available on the scheduled renewal date), or on the date the renewal was due, with one month supply of ART provided, and the result assessed at a follow-up visit after 7-28 days (Figure S1A).*”

And

Line 83: “*For participants randomised to the standard-of-care arm, venous blood was drawn by a research nurse and sent for creatinine and VL testing off-site by the National Health Laboratory Service. VL testing was conducted using the Alinity m HIV-1 VL analyser (Abbott, Chicago, USA). Initially, participants would return to the clinic at a date arranged at their and the healthcare workers convenience, but normally after 7 to 28 days, to be assessed for CCMDD eligibility based on VL and creatinine results (Figure S1A).*”

**Comment 4: Methods:** With regards to outcomes, I wasn’t completely clear on the distinction between CCMDD referall, prescription, ART pick up. My sense is that the clinic writes a referall/prescription and then clients later go and pick up their medications from CCMDD. I also wasn’t sure how this relates to the ART supply a client may have on hand (e.g., some places align the provided drug supply with the next pharmacy appointment) and what all the different point where client becomes at risk for having a lapse in ART.

**Response:** We have now tried to explain this more clearly:

Line 39: **“***Renewed CCMDD prescriptions would again be for six months of ART, with the first two or three months provided at the referral clinic visit, and subsequent ART collected from pick-up points (Figure S1A)* *including private pharmacies, community group venues and smart lockers known as Pele-Boxes, from which patients can retrieve pre-packed medication using a pin code sent to their phone.^12^***”**

**Comment 5:** Results: I might try to report the secondary outcomes essentially in cascade order. Time to VL results, time to prescription, time to ART pick up.

**Response:** We have changed the order as suggested

**Comment 6:** The fact that so few people had CCMDD pick up by 12 weeks made me question if I understood the standard procedures well. Is it that at the time of VL check people are still given 2 to 3 months of medicine…so the main concern with POC vs. SOC VL measurements is the time to getting a result and the need for second visit, rather than a real risk of ART lapse. I initially interpreted CCMDD dormancy as akin to a treatment interruption but perhaps that is not the case. Hence, the comments for clarity in the methods (I do now see some/much of this is clearer with the Figure in the supplement…but would be good to put in main text as the understanding workflow key for interpreting results).

**Response**: Thank you for this, we hope this is now clearer with the added details below and more references to the supplementary figures.

Line 39: **“***Renewed CCMDD prescriptions would again be for six months of ART, with the first two or three months provided at the referral clinic visit, and subsequent ART collected from pick-up points (Figure S1A)* *including private pharmacies, community group venues and smart lockers known as Pele-Boxes, from which patients can retrieve pre-packed medication using a pin code sent to their phone.^12^***”**

We also highlight in the introduction that the major issue is the need for two visits to the clinic, and clients remaining in clinic care, as opposed to treatment interruption:

Line 97: “*VLs are a potential health systems barrier to enrolment and continuation in CCMDD, as testing is conducted at centralized laboratories, meaning clients generally have to attend a clinic twice for a blood draw and then review of results, with results sometimes being delayed or lost (Figure S1A).^8^ This can lead to delays in renewal of CCMDD prescriptions, and increased numbers of dormant clients continuing to attend clinics rather than collecting ART in the community.*”

**Comment 7:** Do SOC procedures make it normal for a CCMDD pick up to be later? E.g., receive 1 month until VL results come back, and then 3 months at the VL check visit…so you don’t go to CCMDD again for 4 months (vs. maybe 3 months if you had POC VL).

**Response:** This would vary by SOC healthcare provider, but often the client would be asked to come back early for their viral load, so that the result was ready on the day the prescription renewal was due, which we have tried to explain below:

Line 32: “*Every 12 months, an annual viral load would be taken, either a few days earlier than the CCMDD prescription renewal was due (so that the result was available on the scheduled renewal date), or on the date the renewal was due, with one month supply of ART provided, and the result assessed at a follow-up visit after 7-28 days (Figure S1A).*”

**Comment 8:** Would be nice to see all secondary outcomes by before and after guideline change in supplement.

**Response:** We have added a new supplementary table (Table S1) with the secondary outcomes by before and after the guideline change.

**Comment 9:** I wasn’t clear why there would be no differences in CCMDD prescriptions but a difference in actually receiving VL (as one should be contingent on other). Although there weren’t big gaps in CCMDD, there does seem to be a big gap in the receipt of VL results, and unclear what this means. There is likely relevance even if it doesn’t end up impacting CCMDD pathway.

**Response:** Wethink this was due to the guideline change, as before the guideline change the median time to VL result in the SOC arm was similar to median time to CCMDD referral (8 days IQR 7 to 21, and 7 days IQR 8 to 21 respectively). After the guideline change, there was no requirement for VL results to be reviewed before CCMDD prescription renewal, so there was no longer any relationship between the two.

**Comment 10:** Might be interesting to understand the differences in what happened among those who had elevated VLs (e.g., time to EAC, suppression, back to CCMDD). The numbers are likely quite small but could warrant a reference as this is somewhere else I might imagine POC VL could have impacts.

**Response:** Thank you for this suggestion. There were so few high VLs that we have not explored this here, but have done a separate study looking at this which we mention and cite (ref 29) below:

Line 79: *“A randomised controlled trial in adults with HIV who had recently initiated ART found point-of-care VL testing was associated with better 12-month viral suppression and retention-in-care,^24^ but other trials in adults,^25^ children,^26^ pregnant women,^27^ adolescents^28^ and people with viraemia,^29^ have not found an impact on viral suppression and retention-in-care.”*

**Comment 11:** The larger analysis with Tier data is clearly related, but seems a little out of place. It seems like it could stand on its own as a paper (and the trial could easily as well). I think it might fit better or be more informative if the exact same type of analyses were performed in this wider dataset before and after the guideline change. In that way, it sort of serves as an alternative control arm that may not be effected by Hawthorne effect of being in a trial.

Even without this, I think would be important to see the before and after guideline change results (rather than just the after). Additionally, I think you would want to include outcomes among everyone in CCMDD that was due for VL rather than also restrict to those who also got CCMDD prescription. I think this would give a broader picture of the guideline change (perhaps more appropriate people in CCMDD, but also more inappropriate…other gaps will probably become evident).

**Response:** Thank you for this suggestion which we have considered carefully. We agree that the larger analysis using routine data, and including results before and after the guideline change and including everyone in CCMDD due a VL, is worthy of a separate paper. We had not done this initially as we did not have enough follow-up time to analyse subsequent outcomes. Now that more follow up time has accrued, we plan the above analysis as a separate paper. We have not removed these initial preliminary results from the current paper as they provide important context, but now state in the discussion:

Line 371: “*Our analysis did not take into account all people in CCMDD who were due a viral load, nor compare before and after the guidelines within routine care, and so we plan a separate, more complete analysis with more follow-up data to better assess the overall impact of this guideline change and whether it improved CCMDD prescription renewal, and its impact on management of viraemia in CCMDD in routine care.*”

**Comment 12:** Discussion: For summary, I would also include the important “null” results. My main takeaway honestly was that people did similarly well for the outcomes we probably care about most (i.e., potential lapse in ART), but there are still other operational and client-associated benefits with regards to receiving the VL results, not having to make additional visit. Although 3 week outcome was selected as primary (and there is some obligation to focus on it in reporting), I don’t think it really provides the appropriate overall story and would try to balance those tensions (For me, articulating the clearest narrative is most important even if it sometimes at odds with the “rules” of clinical trial reporting). That being said, the rest of the discussion provides an excellent narrative synopsis of the meaning of the totality of results.

**Response:** We now say:

**Line 347: *“****In this randomised controlled trial we found that point-of-care testing reduced the number of people with a delayed CCMDD prescription renewal of three or more weeks, who are defined as ‘dormant clients’ in the CCMDD programme, but did not improve overall retention-in-care or time to CCMDD ART collection. Point-of-care testing also reduced the time to receipt of VL results, and the number of clinic visits, and associated travel costs, required by participants for CCMDD prescription renewal.”*

**Comment 14:** Is there anything to be said regarding cost of implementing vs. cost (and time) savings from client end?

**Response:** We do not have data from this study on implementation costs versus complete client costs, but now say:

**Line 411: “***Health economic studies that account for costs of point-of-care viral load implementation, and potential healthcare system and client cost savings, are also required.*”

Sincerely,

**Dr Jienchi Dorward** (on behalf of all authors)

**MBChB, BSc, MSc, Dip HIV Man**

Academic Clinical Lecturer, University of Oxford

Specialist Primary Care Physician

CAPRISA Honorary Associate Scientist
